# Efficacy of a low-FODMAP diet in children with irritable bowel syndrome and functional abdominal pain–not otherwise specified: a randomized controlled trial with early termination

**DOI:** 10.3389/fped.2026.1809053

**Published:** 2026-03-18

**Authors:** Agata Stróżyk, Andrea Horvath, Anna Kozłowska-Jalowska, Jane Muir, Hania Szajewska

**Affiliations:** 1 Department of Paediatrics, Medical University of Warsaw, Warsaw, Poland; 2 Department of Gastroenterology, Monash University, Melbourne, VIC, Australia

**Keywords:** children, diet, functional abdominal pain disorders, irritable bowel syndrome, low-FODMAP

## Abstract

**Objective:**

Evidence supporting the effectiveness of a low fermentable oligosaccharides, disaccharides, monosaccharides, and polyols (low-FODMAP) diet in treating functional abdominal pain disorders remains limited. We aimed to assess the efficacy of a low-FODMAP diet compared with a regular diet for management of children with irritable bowel syndrome (IBS) and functional abdominal pain–not otherwise specified (FAP-NOS).

**Methods:**

This randomized controlled trial enrolled children aged 8–18 years with IBS or FAP-NOS, diagnosed using the Rome IV criteria. Participants were assigned to a 4-week low-FODMAP diet or a regular diet. The primary outcome was the proportion of responders, defined as those with a ≥30% reduction in abdominal pain, corresponding to at least the Reliable Change Index (≥25 mm change on visual analog scale), over the 4-week intervention.

**Results:**

The trial was terminated early due to recruitment challenges. Of the 74 originally planned participants, 42 (57%) were randomized, and 38 (51%) received the intervention, with 19 in each group. In the available case analysis (including only participants with outcome data at week 4), there was no difference in the proportion of responders between the low-FODMAP (7/13) and regular diet (5/13) groups at week 4 [relative risk 1.4 mm, 95% confidence interval (CI), 0.6–3.3]. In the secondary outcomes, only a greater mean reduction in abdominal pain intensity was observed in the low-FODMAP diet group at week 4 (mean difference = −20.8 mm, 95% CI, −40.0 to −1.6; 26/38). Adverse events were rare in both groups (*n* = 4).

**Conclusions:**

This early-terminated RCT did not demonstrate a significant benefit of the low-FODMAP diet in increasing the proportion of responders among children with IBS and FAP-NOS. However, due to early termination and missing data, the findings should be interpreted with caution. Nonetheless, the study provides insights into challenges in conducting controlled dietary intervention trials in children (e.g., the demanding nature of a highly restricted dietary protocol and practical issues related to school settings and caregiver involvement), which may inform the design of future studies.

**Clinical Trial Registration:**

https://www.ClinicalTrials.gov Identifier: NCT04528914.

## 1 Introduction

Functional abdominal pain disorders (FAPDs), also known as disorders of gut–brain interaction, are chronic conditions characterized by clusters of gastrointestinal symptoms in the absence of organic or biochemical causes (1–3). According to the Rome IV criteria (1), FAPDs are subcategorized into four subtypes: irritable bowel syndrome (IBS), abdominal migraine, functional dyspepsia, and functional abdominal pain–not otherwise specified (FAP-NOS). The overlap in clinical presentation and proposed treatment is particularly substantial for IBS and FAP-NOS (4). FAPDs affect approximately 11.7% of children aged 4–18 years worldwide, with IBS accounting for 5.8% of cases and FAP-NOS being the least prevalent (1.2%) (5, 6).

The lack of effective, evidence-based treatment strategies for FAPDs in children often drives parents and caregivers to consider alternative approaches, including dietary interventions. However, the evidence supporting dietary management of IBS and FAP-NOS in children remains limited (7). One group of potential dietary triggers includes fermentable oligosaccharides, disaccharides, monosaccharides, and polyols (FODMAPs) (2). A low-FODMAP diet limits intake of short-chain carbohydrates that are poorly absorbed in the small intestine and rapidly fermented by colonic microbiota (8). This fermentation process results in the production of short-chain fatty acids and gases, leading to intestinal distention and stimulation of mechanoreceptors, which may contribute to abdominal pain and bloating in children with FAPDs (3).

Despite increasing clinical use, the efficacy of the low-FODMAP diet in children with FAPDs has not been clearly established (2, 8, 9). Three systematic reviews have concluded that there is insufficient evidence to support or refute its use in children with FAPDs (7, 10, 11). Consequently, both the European Society of Gastroenterology, Hepatology and Nutrition (ESPGHAN) and a consensus statement by four Italian scientific societies (Italian Society of Gastroenterology, Hepatology and Pediatric Nutrition; Italian Society of Pediatrics; Italian Society of Gastroenterology and Endoscopy; Italian Society of Neurogastroenterology and Motility)(9) do not recommended the routine use of the low-FODMAP diet in children (8, 9). However, according to the ESPGHAN Position Paper, this approach may be considered in a carefully selected subgroup of children with IBS—particularly those at low risk for developing eating disorders, who accept the low-FODMAP alternatives and have access to a qualified pediatric dietician (8). In the present trial, we aimed to assess the efficacy and safety of a low-FODMAP diet compared with a regular diet in the management of children with IBS and FAP-NOS.

## 2 Material and methods

This was a single-center, parallel-group, randomized, and controlled superiority trial conducted at the Department of Paediatrics, Medical University of Warsaw. The study protocol was registered in the ClinicalTrials.gov database (NCT04528914) and published in a peer-reviewed journal prior to the enrollment of the first patient (12). The trial adhered to the Consolidated Standards of Reporting Trials (CONSORT) 2010 guidelines (13). Recruitment was delayed due to the COVID-19 pandemic and ultimately took place between January 2023 and November 2024.

### 2.1 Participants

Children aged 8–18 years with IBS or FAP-NOS diagnosed according to the Rome IV Criteria were included. Participants were required to have a baseline average pain intensity of at least 30 mm on a 100-mm visual analog scale (12).

Given the unsatisfactory recruitment rate after study initiation, the exclusion criterion of overweight or obesity was removed. This decision was supported by national epidemiological data indicating that up to 29% of girls and 36% of boys aged 7–9 years in Poland are affected by overweight or obesity (14).

In addition, because the study design involved the provision of full-day meal boxes by a contracted food service provider operating within a limited geographic area, enrollment was restricted to children residing in Warsaw.

### 2.2 Randomization and allocation

Participants were randomly assigned to one of two groups: the low-FODMAP diet or a regular diet, with the intervention lasting 4 weeks. A block randomization method (using variable block sizes of 4 and 2) in a 1:1 ratio was applied using a computer-generated randomization sequence (StatsDirect statistical software England: StatsDirect Ltd. 2013). The sequence was prepared by an independent individual with no clinical involvement in the trial. Allocation concealment was maintained through the use of opaque, sealed, and sequentially numbered envelopes.

### 2.3 Masking

Following group assignment, a research dietitian developed individualized meal plans for each participant according to the allocated diet. The study was designed with quadruple blinding, in which participants, their caregivers, and two study investigators (AH, AK-J) were blinded to group assignments until completion of the intervention. Unblinding was permitted only after the intervention period had ended or upon participant withdrawal, to guide further clinical management. For ethical reasons, participants and caregivers were informed prior to enrollment about the general nature of the two dietary interventions being compared. However, the research dietitian (AS)—who not only developed the diets but also enrolled participants, collected data, and interpreted the study results—remained unblinded throughout the study.

After the end of the 4-week intervention, each participant was asked to indicate whether they believed they had received the low-FODMAP or regular diet (15). No prespecified cut-off was defined to assess blinding success. The catering company was informed of the assigned diet for each participant; however, no potentially suggestive information (i.e., no information on the ingredients of delivered meals) was included with the delivered meal boxes.

To minimize the risk of unblinding, meals provided to both groups were matched as closely as possible. In the regular diet group, selected low-FODMAP products (i.e., gluten-free bread or snacks) were included to reduce the likelihood of participants correctly identifying their group allocation. Participants and caregivers were instructed not to attempt to identify the type of diet using external resources and, if they suspected the allocation, not to disclose their assumptions to the child.

The independent statistician conducting the data analysis remained blinded until the final analysis was completed.

### 2.4 Intervention

This study was designed as a home-based feeding trial, in which all meals were provided to participants for home consumption over a period of 4 weeks (16). Diets in both the low-FODMAP and regular diet groups were matched to the participants' habitual diets in terms of total energy, fat, protein, carbohydrates, and dietary fiber (12). The intervention was fully described in the published protocol (12). Initially, we planned to verify each meal plan developed by the research dietitian for FODMAP content with the Monash FODMAP Calculator (https://www.monashfodmapcalculator.com.au/) (12). However, this platform provides FODMAP content reports only after approval by the Australian research team (usually ≥24 h after submission). It does not allow for real-time assessment during meal plan development using dietary analysis software, nor does it support tailoring the meal plan to the participant's habitual FODMAP intake.

Therefore, we used the Monash FODMAP Calculator only to estimate participants' baseline FODMAP intake (including excess fructose, lactose, sorbitol, mannitol, fructans, galactooligosaccharides, and total oligosaccharides), rather than to tailor individual meal plans.

As in previous RCTs (17), each FODMAP-restricted meal plan was developed using the low-FODMAP content portion sizes reported in the Monash FODMAP App (an official database provided by Monash University). A Monash FODMAP-trained dietitian created the plans, following standard clinical practice. The app's food grading system was applied: Only foods with portion sizes classified as low-FODMAP (green rating) were included in the meals (18).

In the control group (regular diet), meal plans were developed to reflect each child's usual diet and FODMAP intake, based on the 3-day food record. Participants received either four or five meals per day, depending on their typical eating habits.

A complete all-day box diet was delivered each morning to the participants’ residence by a designated food service provider. All caregivers received a list of permitted snacks. In both groups, snacks were selected for their low FODMAP content (i.e., portion of nuts, gluten-free biscuits or crackers, eggs, popcorn, low-FODMAP fruits) to minimize the risk of unblinding. If participants or caregivers had any questions related to the assigned diet, dietary support was made available throughout the intervention period.

### 2.5 Procedure

At the initial visit, all participants were diagnosed with IBS or FAP-NOS by a pediatric gastroenterologist. Any anthropometric measurements were performed by a trained nurse or dietitian. BMI-for-age *z*-scores were calculated using the WHO AnthroPlus software (version 1.0.4.) and/or WHO growth reference charts (*z*-scores).Percentiles were determined using the Polish OLA/OLAF growth charts (19). During the initial dietary consultation, each child and caregiver received both oral and written information regarding the study. The study design, including risks and expected benefits, was discussed in detail during a face-to-face or online meeting. Written informed consent was obtained from all participants' caregivers before enrollment.

Prior to enrollment, participants completed a baseline questionnaire reflecting symptoms and functioning over the preceding week. The included questions addressed abdominal pain intensity and frequency, stool consistency, school absenteeism, and parental work absenteeism. Participants or their caregivers also completed the Gastrointestinal Symptom Rating Scale (GSRS), the KIDSCREEN-10 index, and the World Health Organization Five Well-Being Index (WHO-5). All questionnaires were then repeated weekly by the participants during the 4-week intervention. Baseline physical activity was assessed using a screening tool for moderate-to-vigorous physical activity (MVPA). During the intervention period, participants were asked to complete a daily subject diary. Each day, the child or caregiver reported the percentage of each meal consumed, the characteristics of any additional snacks, and diet tolerance assessed using a 100-mm VAS.

Any concerns related to the dietary intervention (e.g., issues with meal delivery or adverse symptoms) were documented in the subject diary and reported to the research dietitian. All subjects, after discontinuation or completion of the trial, were encouraged to continue management of their FAPD under the care of the study center.

Dietary adherence was assessed by the research dietitian based on direct interviews with the children or caregivers and analysis of subject diary data. Adherence was calculated as the percentage of consumed meals and snacks.

### 2.6 Outcome measures

The primary outcome was prespecified as the change in abdominal pain intensity, reported by the patient or caregiver using the 100-mm VAS. Responders were defined as those who achieved weekly reduction in abdominal pain intensity of at least 30% at week 4 compared with baseline, with an absolute reduction of at least 25 mm, corresponding to the Reliable Change Index adopted for this sample (20, 21). According to the CONSORT Patient-Reported Outcomes (PRO) Extension (22), the number of missing data points and reasons for the same were also reported.

Secondary outcomes were based on the standardized core outcome set (23). The number of children with reduction in abdominal pain intensity of at least 25 mm from baseline at weeks 1, 2, and 3 was reported. Other secondary outcomes included changes from baseline to weeks 1–4 in the following:

- mean abdominal pain intensity,
- stool consistency [number of responders (12)],
- abdominal pain frequency (decline, increase or no change),
- GSRS total score,
- KIDSCREEN-10 index total score,
- WHO-5 total score,
- number of child's days of absence at school (percentage),
- number of parental days of absence at work (percentage),
- compliance (percentage), and
- tolerance (mean).

Moreover, the number of children experiencing any adverse events during the 4-week intervention was reported.

Due to recruitment challenges and parental preference to minimize onsite visits, anthropometric measurements were not repeated at week 2 as originally planned. Therefore, BMI-for-age *z*-score was assessed as change from baseline at week 4.

### 2.7 Sample size calculation

Based on the recommendation of the Rome Foundation Pediatric Subcommittee for clinical trials in children with IBS (20), we assumed a 30% percentage point difference in the proportion of responders between the interventional and control groups as the clinically meaningful effect size for the primary outcome. To detect this difference with 80% power, a two-sided 5% significance level, and allowing for a 20% anticipated dropout rate, we estimated that 74 children (37 subjects per group) would be required. Sample size calculations were performed using StatsDirect (StatsDirect Ltd. 2013, http://www.statsdirect.com).

This trial was terminated early—after enrolling 42 subjects—due to recruitment challenges and adherence issues. The lack of efficacy was not a reason for early termination, given the underpowered nature of the study.

### 2.8 Data analysis

Descriptive statistics were used to present the participants' baseline characteristics. Nominal variables are presented as the number of patients (*n*) and percentages (%). Continuous variables are reported as means and standard deviation (SD) or medians with interquartile range (Q1; Q3), as appropriate (depending on data distribution). The normality of distribution was assessed using the Shapiro–Wilk test, along with skewness and kurtosis values. All tests were two-tailed with a significance level of *α* = 0.05. For primary and secondary outcomes, mean or median differences (MD) between groups, or relative risks (RR), were calculated, each with corresponding 95% confidence intervals (CI). The number of participants who received the intervention and the number analyzed for each outcome were also reported (22).

Because of the high rate of missing data, an available case analysis (ACA) analysis was performed, including all randomized participants who received the intervention for at least 1 day with available data. In addition, a per-protocol analysis, excluding participants who did not complete the 4-week dietary intervention or who failed to return any subject diaries, was considered. However, as there was no change in the number of analyzed participants for primary outcomes, this was not reported.

Sensitivity analysis was performed only for the primary outcome in ITT dataset. The missing data were handled using multiple imputation under the missing-at-random assumption, generating 20 datasets (using mice and miceafter packages in R software). For binary outcomes, frequencies were averaged across imputations, with percentages derived further based on given number of patients in each group. Risk ratios with 95% confidence intervals were pooled across imputations, and Wald *p*-values were derived from the log risk ratio.

Although logistic regression including three potential confounders (age, sex, and IBS subtype) was prespecified in the study protocol, given the limited sample size and the absence of significant baseline differences between groups for these variables, such an analysis would be challenging and could be underpowered; therefore, it was not performed. While baseline BMI and excess fructose intake showed some imbalance, the limited sample size precluded reliable adjustment through regression modeling.

All statistical analyses were conducted using R version 4.2.1. (R Core Team, 2022. R: A language and environment for statistical computing. R Foundation for Statistical Computing, Vienna, Austria).

## 3 Results

### 3.1 Study characteristics

**Figure 1:**
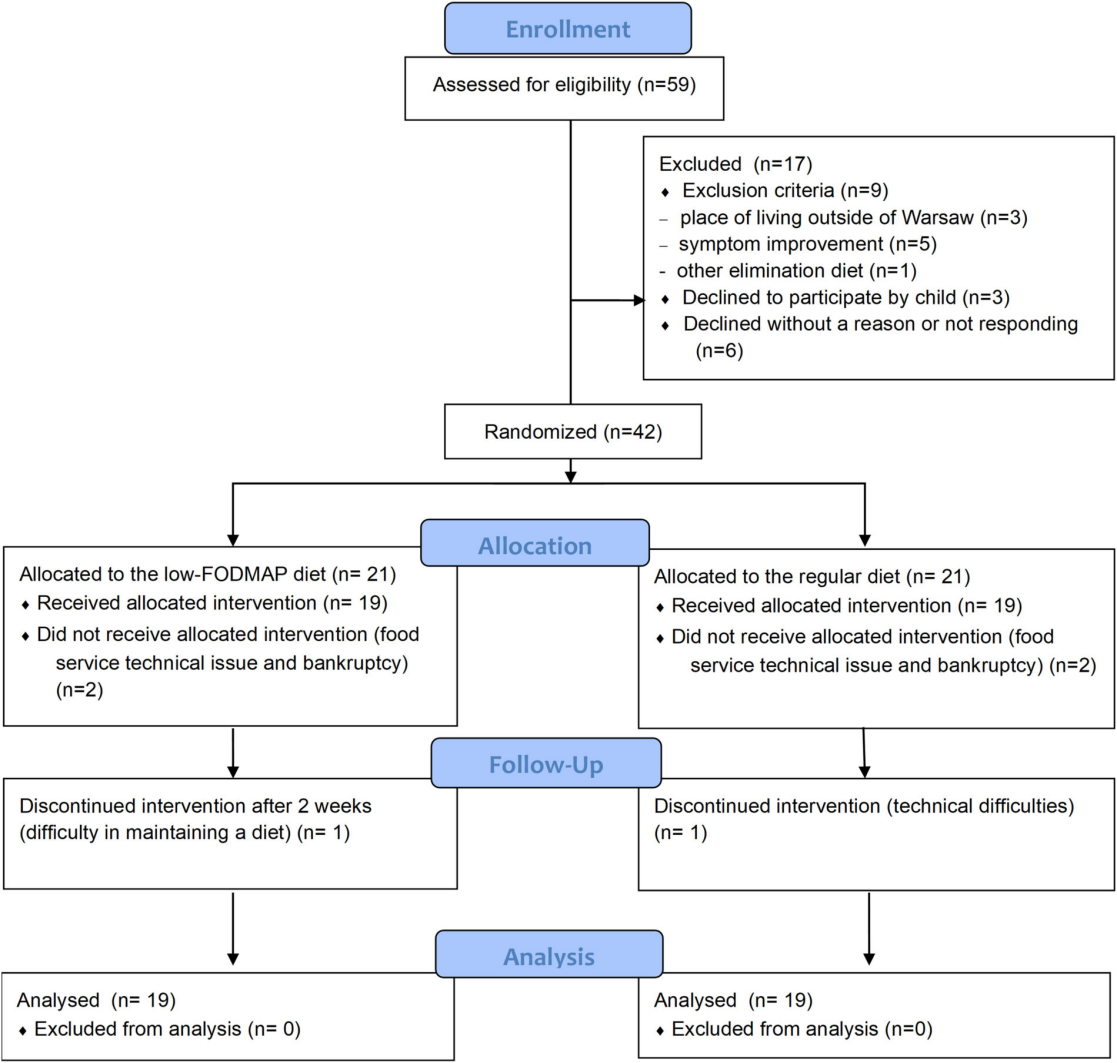
Participants selection. PRO, patient reported outcomes.

A flow diagram of the trial is presented in Figure 1. Enrollment was originally scheduled to begin in January 2020; however, due to the outbreak of the COVID-19 pandemic in December 2019, recruitment was postponed. Eligible patients were ultimately recruited between 1 February 2023 and 31 December 2024.

A total of 42 participants were randomized, of whom 38 (53% girls; mean age: 13.3 years) received the allocated intervention. All randomized participants were included in the intention-to-treat analysis. Four children did not receive intervention due to issues with the catering provider. Two children discontinued intervention—one after 2 weeks due to difficulty adhering to the diet, and another after 3 days because of challenges with reheating meals at school. An additional five children discontinued the intervention prematurely due to problems with the food service provider (one after 3 weeks and four after 2 weeks).

Recruitment and retention were also impacted by pandemic-related disruptions. Changes in school infrastructure and catering logistics introduced during and after the COVID-19 pandemic limited the feasibility of consistent meal provision. Furthermore, the prolonged period of remote learning and social isolation may have reduced families' willingness to engage in structured, daily interventions requiring external coordination and active caregiver involvement.

Baseline characteristics of participants included in the analysis were similar between the two groups (Table 1). The only difference was observed in the baseline median BMI: low-FODMAP diet group (median: 20.0; Q1–Q3: 18.4; 21.8) versus regular diet group (median: 17.7 Q1–Q3: 15.9; 20.0). Mean energy, protein, fats, carbohydrates, and fiber intakes were comparable between groups (Table 2). Baseline FODMAP intake was also similar across most subtypes; however, median intake of excess fructose was higher in the low-FODMAP diet group compared with the regular diet group.

**Table 1:** Characteristics of study groups.

Variable	Low-FODMAP diet (*n* = 19)	Regular diet (*n* = 19)	*p*
Age (years), mean ± SD	13.8 ± 2.8	12.9 ± 3	0.356
Sex, *n* (%)			
Female	10 (52.6)	10 (52.6)	>0.999
Male	9 (47.4)	9 (47.4)	
MVPA (day/week), mean ± SD	3.9 ± 1.4	4.5 ± 2	0.349
Baseline abdominal pain intensity (measured on a 100-mm VAS) (mm), mean ± SD	55.3 ± 18.1	49.7 ± 15.8	0.322
Anthropometric measures			
Weight (kg), mean ± SD	58.7 ± 19	49 ± 22.3	0.159
Height (cm), mean ± SD	163.7 ± 14.1	157.2 ± 18.9	0.241
BMI, median (Q1;Q3)	**20 (18.4;21.9)**	**17.7 (15.9;20.0)**	**0.049^2^**
BMI for age percentile (OLA/OLAF) [percentiles] baseline, mean ± SD	57.1 ± 31.2	40.6 ± 31.2	0.113
BMI-for-age z-score (SD), mean ± SD	0.3 ± 1.2	−0.3 ± 1.3	0.151
FAPD subtypes			
FAPD subtype, *n* (%)			
FAP-NOS	10 (52.6)	8 (42.1)	0.745
IBS	9 (47.4)	11 (57.9)	
IBS subtype, *n* (%)			
IBS-C	5 (55.6)	4 (36.4)	0.811^1^
IBS-D	4 (44.4)	6 (54.5)	
IBS-M	0 (0.0)	1 (9.1)	

Groups compared with chi-square test or Fisher exact test^1^ for nominal variables and with *t*-test or Mann–Whitney *U*-test^2^ for continuous variables.

BMI, body mass index; FAPD, functional abdominal pain disorders; FAP-NOS, functional abdominal pain–not otherwise specified; IBS, irritable bowel syndrome; IBS-C, irritable bowel syndrome with predominant constipation; IBS-D, irritable bowel syndrome with predominant diarrhea; IBS-M, irritable bowel syndrome with mixed bowel habits; MVPA, moderate-to-vigorous physical activity; Q1, lower quartile; Q3, upper quartile; OLA/OLAF, percentiles growth charts for Polish children; SD, standard deviation; VAS, visual analogue scale.

Bold values indicate statistical significance (*p* < 0.05).

**Table 2:** Baseline FODMAP, energy, and nutrients intake.

Variable	Low-FODMAP diet (*n* = 19)	Regular diet (*n* = 19)	*p*
Baseline FODMAP Intake (g per serving of food per sitting)			
Excess Fructose, median (Q1; Q3)	0.3 (0.2; 1.5)	0.1 (0.1; 0.3)	0.013^1^
Lactose, median (Q1; Q3)	1.5 (0.4; 2.9)	0.4 (0.0; 2.1)	0.129^1^
Sorbitol, mean ± SD	0.3 ± 0.4	0.1 ± 0.2	0.059
Mannitol, median (Q1; Q3)	0.0 (0.0; 0.0)	0.0 (0.0; 0.0)	0.573^1^
Fructans, median (Q1; Q3)	0.6 (0.5; 0.7)	0.5 (0.4; 0.9)	0.965^1^
GOS, median (Q1; Q3)	0.1 (0.1; 0.1)	0.1 (0.1; 0.2)	0.569^1^
Total Oligos, median (Q1; Q3)	0.7 (0.6; 0.8)	0.7 (0.6; 1.0)	0.630^1^
Baseline energy and nutrients intake			
Mean energy intake (kcal), median (Q1; Q3)	1,923.0 (1,789.8; 2,225.5)	1,843.0 (1,750.5; 1,930.3)	0.191^1^
Mean protein intake (g), mean ± SD	85.4 ± 23	82.3 ± 21.6	0.671
Mean fats intake (g), median (Q1; Q3)	65.6 (50.9; 80.8)	65.1 (51.2; 74.5)	0.583^1^
Mean carbohydrates intake (g), mean ± SD	265.5 ± 65.5	232.6 ± 41.8	0.074
Mean fiber intake (g), mean ± SD	18.5 ± 5.8	18.1 ± 5.5	0.832

Groups compared with *t*-test or Mann–Whitney *U*-test^1^ for continuous variables.

Q1, lower quartile; Q3, upper quartile; SD, standard deviation.

Following the low-FODMAP diet cut-off values for each sugar (per serving of food per sitting) (18), diets in both groups were high in lactose, fructans, and oligosaccharides. A high intake of sorbitol and excess fructose was observed only in the low-FODMAP diet group. Median mannitol and galactooligosaccharides intakes were very low in both groups.

### 3.2 Primary outcome

In the ACA, no difference was observed in the number of responders between the low-FODMAP diet group (7/13) and the regular diet group (5/13) over the 4-week intervention (RR = 1.4 mm, 95% CI, 0.6–3.3) (Table 3). A substantial rate of missing data for primary patient-reported outcome was noted (12/38; 32%), mainly due to lack of questionnaire return (*n* = 7) and preliminary termination of diet supply because of catering provider bankruptcy (*n* = 5).

**Table 3:** Effect of a low-FODMAP diet on change in abdominal pain intensity (the available case analysis).

Outcome and time point of assessment	Low-FODMAP diet		Regular diet		MD/RR (95% CI)	*p*
	*n*		*n*			
Number of responders over the 4-week intervention (who met prespecified criteria for decrease in abdominal pain intensity from baseline), *n* (%)^a^						
At week 4	13	7 (53.8)	13	5 (38.5)	1.4 (0.6; 3.3)	0.694
Number of responders at week 1, 2, and 3 (who met prespecified criteria for decrease in abdominal pain intensity from baseline), *n* (%)^a^						
At week 1	18	9 (50.0)	14	2 (14.3)	3.5 (0.9; 13.7)	0.061^1^
At week 2	17	9 (52.9)	15	5 (33.3)	1.6 (0.7; 3.7)	0.448
At week 3	9	6 (66.7)	12	6 (50.0)	1.3 (0.6; 2.8)	0.750
Change in abdominal pain intensity from baseline (measured on a 100-mm Visual Analogue Scale) (mm)						
At week 1, median (Q1;Q3)	**18**	**−20.0 (−30.0; −10.0)**	**14**	**0.00 (−10.0; 0.0)**	**−20.0** **(−30.0; −5.0)**	**0** **.** **013^2^**
At week 2, mean ± SD	17	−23.8 ± 24.5	15	−15 ± 28.2	−8.8 (−28.1; 10.4)	0.355
At week 3, mean ± SD	9	−25 ± 21.9	12	−23.3 ± 32.6	−1.7 (−26.6; 23.3)	0.890
At week 4, mean ± SD	**13**	**−29.6 ± 27.7**	**13**	**−8.9 ± 18.6**	**−20.8** **(−40; −1.6)**	**0** **.** **036**

SD, standard deviation; Q1, lower quartile; Q3, upper quartile; MD, mean/median difference between groups calculated as low-FODMAP group minus regular diet group; RR, relative risk between groups calculated as low-FODMAP group vs. regular diet group. Groups compared with chi-square test or Fisher’s exact test^1^ for nominal variables and with *t*-test or Mann–Whitney *U*-test^2^ for continuous variables.

Bold values indicate statistical significance (*p* < 0.05).

a Responders were defined as participants who reported a ≥30% reduction in abdominal pain intensity and a reduction of ≥25 mm on a 100-mm visual analogue scale, over the 4-week intervention.

### 3.3 Sensitivity analysis

In the sensitivity analysis based on ITT dataset, using data after implementing multiple imputation, no difference between the groups was confirmed for the proportion of responders at any follow-up during the 4-week intervention (Supplementary Table S1).

### 3.4 Secondary outcomes

No difference was observed in the number of responders between the low-FODMAP diet and regular diet groups at weeks 1–3 (Table 3). However, this outcome was reported by 84% of participants (32/38) at week 1 and 2, and by only 45% at week 3 (17/38).

A higher reduction in abdominal pain intensity (measured on a 100-mm VAS) from baseline to week 1 was observed in the low-FODMAP diet group compared with regular diet group [median: −20 mm (18/19) vs. −0 mm (14/19); median difference = −20.00 mm; 95% CI, −30.0 to −5.0, 32/38] (Table 2). This difference was also significant at week 4 [mean: −29.6 ± 27.7 vs. −8.85 ± 18.6 mm (13/19 in both groups); MD = −20.8 mm, 95% CI, −40 to −1.6; *p* = 0.036, 26/38]. No significant differences were found between groups for any other secondary outcomes (Supplementary Table S2).

During the 4-week intervention, the mean percentage adherence to the dietary interventions was comparable between groups (mean ± SD: 73.9 ± 15.9% in the low-FODMAP diet group vs. 70.3 ± 16.2% in the regular diet group, 32/38).

No significant differences were found in tolerance of the intervention [reported by child in a 100-mm VAS; 70.8 ± 20.0 mm in the low-FODMAP diet group (19/19) vs. 67.9 ± 16.3 mm (14/19) in the regular diet group; 33/38].

The number of compliant participants (defined as consuming ≥80% of the provided diet) was similarly low in both groups: 7 of 17 (41.2%) in the low-FODMAP diet group and 5 of 15 (33.3%), in the regular diet group (RR = 1.2, 95% CI, 0.5–3.1; 32/38).

The number of participants with adverse events was equal in both groups (two children in each group; RR = 1, 95% CI, 0.2–6.0; 37/38). Three children developed constipation, which was successfully managed with macrogol. One child reported a 2-kg weight loss after 2 weeks of intervention; therefore, the caloric content of the diet was increased.

At the end of the 4-week period, the percentage of participants who reported successful blinding was similar between the low-FODMAP diet group (19/19) and the regular diet group (17/19) (63.2% vs. 88.0%; RR = 0.72; 95% CI, 0.49–1.05; *p* = 0.128).

### 3.5 Follow-up

In the low-FODMAP diet group, eight of 19 children (42%; seven responders and one non-responder) began FODMAP reintroduction. In the regular diet group, five participants consulted the dietitian to initiate the low-FODMAP diet (including two placebo responders); among these, only one adopted a lactose-free diet. An additional two children (one in each group) expressed interest in initiating or continuing the low-FODMAP diet, but only if it was provided by a food service.

In four children in the low-FODMAP diet group (including one responder) and six in the regular diet group (including one placebo responder), alternative treatments—such as probiotics, psychologic consultation, and/or simple dietary modifications (i.e., regular meals, reduced intake of ultra-processed foods, lower portion sizes)—were recommended.

Eight participants (four in each group) declined any further treatment, primarily due to symptom improvement. Five children were lost to follow-up (two in the low-FODMAP diet group and three in the regular diet group).

## 4 Discussion

### 4.1 Summary of findings

This RCT was terminated early due to recruitment challenges and adherence issues. Although, there was no difference in proportion of responders between the low-FODMAP diet and regular diet groups, the early termination and small sample size limit the generalizability of these findings. Therefore, we cannot exclude the possibility that the observed results reflect insufficient power rather than a true lack of effect. Despite the small sample size, reporting these findings is important to ensure transparency, highlight the practical challenges of dietary intervention trials, and inform the design of future studies.

In this early-terminated RCT, a 4-week low-FODMAP diet did not demonstrate a significant benefit in the proportion of responders (defined as a ≥30% decrease in abdominal pain intensity, equivalent to at least 25 mm) compared with a regular diet. However, the low-FODMAP diet group showed a greater mean reduction in abdominal pain intensity from baseline to both week 1 and week 4. These findings are limited by a substantial amount of missing data, which may reflect the demanding nature of the dietary protocol and practical challenges related to school settings and caregiver involvement.

No benefits of the low-FODMAP diet were observed for the other secondary outcomes. Mean adherence to the dietary intervention was moderate in both groups, but the number of compliant participants (consuming ≥80% of the provided diet) was low in both arms.

Successful blinding was achieved in most of participants, although slightly less frequently in the low-FODMAP diet group. Notably, challenges related to dietary implementation in a post-pandemic context may have contributed to both reduced adherence and the extent of missing data.

Finally, the extent of missing data may reflect the still low public awareness of the importance of clinical research. In some cases, participants who experienced symptom improvement discontinued data reporting despite repeated reminders.

### 4.2 Comparison with other studies and systematic reviews

The lack of a significant difference in the proportion of responders between the low-FODMAP diet and regular diet in our study remains difficult to interpret.

The effectiveness of any dietary intervention depends on adherence. Consistent with our findings, another Polish RCT (17) assessing the effects of the low-FODMAP diet versus dietary modifications based on NICE guidelines in children with functional abdominal pain reported no significant differences in adherence between groups at weeks 1, 3, and 4. In adults, studies have demonstrated a positive correlation between higher adherence to the low-FODMAP diet and greater improvement in gastrointestinal symptoms (24). The moderate adherence found in our study could explain the limited benefit of the low-FODMAP diet. These findings also underscore the challenges of achieving full compliance with the low-FODMAP diet in pediatric populations, even if all meals are provided and the intervention is individualized.

The low-FODMAP diet involves limitation or exclusion of foods from nearly all food groups (25). In pediatric populations, such restrictions may potentially impact growth and development (26). Some children may experience fatigue and reduced dietary satisfaction during a 4-week restrictive diet, although this was not systematically assessed. Future studies should evaluate the psychological and behavioral impact of dietary interventions. So far, no studies have assessed the relationship between adherence to the low-FODMAP diet and the risk of developing eating disorders and feeding difficulties in children. However, in adults, higher adherence to the low-FODMAP diet has been associated with an increased frequency of eating disorder behaviors (27).

In response to these concerns in vulnerable populations, a step-up approach—also referred to as the “FODMAP-gentle” method—has been proposed (25). This strategy involves initially restricting only one FODMAP subgroup or a limited number of high-FODMAP foods, with further restrictions introduced only if symptom relief is insufficient (26, 28, 29). Although this simplified approach has not yet been assessed in pediatric populations, a less restrictive version of the low-FODMAP diet may be a practical and reasonable alternative to consider (25, 26).

The regular diet was selected as the control diet based on the assumption that it would have no symptomatic effects (15). However, some impact was observed. In certain cases, the provision of fully delivered, all-day meals resulted in improved regularity, better portion control, reduced intake of ultra-processed foods, and decreased frequency of fast-food consumption.

In another RCT (30), which analyzed only the low-FODMAP diet arm, the authors reported that children with FAPDs had nutritionally inadequate diets at baseline. Following the introduction of the low-FODMAP diet, modest improvements were noted in both the intake of certain micronutrients and overall diet quality (30). These findings may help explain the similar number of responders in both the low-FODMAP diet and regular diet groups in our study. In populations with FAPDs, the risk of a placebo response in clinical trials is high, regardless of investigated treatment (15, 31). In our study, the considerable number of responders in the regular diet group may also be partially explained by a placebo effect. To our knowledge, the placebo response rate in pediatric FAPDs has been reported in only one short-term (3-day) RCT (32). In that study, 33.3% of children responded to a typical American childhood diet, which is a proportion comparable to the 38.5% of responders in the regular diet observed at 4 weeks in our study.

Interestingly, one cross-over open-label RCT involving children and adolescents with FAP or IBS (*n* = 30) demonstrated that a 3-week open-label placebo period led to a reduction in abdominal pain and a decreased need for rescue medication compared with a 3-week control period (33). The role of open-label placebo in managing IBS/FAP-NOS in children remains under debate. Nevertheless, in clinical trials, the implementation of a run-in phase using the intervention of interest has been proposed as a strategy to identify potential placebo responders and thus minimize this effect (34). However, the extended duration of a highly restrictive diet may impose additional burden on both children and parents, and increase cost. For these reasons, a run-in phase was not incorporated into our study design.

### 4.3 Strengths and limitations

Despite early termination, this study provides valuable insights into the challenges associated with dietary intervention trials in children. It was designed as a home-based controlled feeding trial, which is considered the gold standard for generating high-quality evidence on the efficacy of dietary interventions (16).

The provision of all meals to participants enhanced the accuracy of dietary exposure, reduced participant burden by eliminating the need for meal preparation, and enabled blinding of the dietary treatment (16, 35).

To our knowledge, this is the first RCT assessing the efficacy of the low-FODMAP diet in children using well-defined outcomes following the core outcome set for clinical trials in pediatric FAPDs (23, 35). It is also the first pediatric study to assess the success of blinding in a dietary intervention. Given the widespread availability of information about the low-FODMAP diet, particularly among adolescents, there is a legitimate risk that participants may recognize their group allocation (17). Therefore, it is essential that dietary trials report whether participants correctly identified their group allocation (15). In contrast to one trial in adults with IBS (*n* = 38), where 83% of participants correctly recognized allocation to the low-FODMAP diet (36), our findings support the feasibility of successful blinding in pediatric low-FODMAP diet trials.

Another strength of this trial was the detailed assessment of baseline participants' FODMAP intake, which is rarely reported in pediatric studies. These data may support the development of simplified or stepwise low-FODMAP diet approaches. Future research assessing habitual FODMAP intake in larger pediatric populations could further guide clinical implementation.

The main limitation of this study is the small sample size, which may introduce selection bias and limit generalizability. However, this reflects the logistical and financial challenges inherent to controlled feeding trials. Such trials are typically complex and resource-intensive, often enrolling fewer than 50 participants (16). Among existing pediatric trials of the low-FODMAP diet, sample sizes ranged from 13 to 84 participants (10, 37). Trials with sample sizes at the higher end of this range [involving 60 (38) to 84 (37)] were typically dietary counselling studies, in which written instructions were provided rather than full meal provision (34). These designs are generally easier to implement than feeding trials but offer less control over dietary exposure and adherence (34). Despite its limited sample size, our study contributes valuable insights from a rigorously controlled setting that complements findings from larger, less intensive trials.

A major logistical challenge in our study was securing a commercial food service provider capable of delivering individualized diets for children. An alternative approach—preparing meals within the research team—is only feasible in a limited number of specialized nutrition research centers worldwide (32, 34, 36).

Another important limitation was the high rate of missing data. Despite repeated reminders and follow-up efforts, many caregivers did not return the complete 4-week symptom diaries. In our trial, diaries were completed in either paper form or electronic form via email, which may have contributed to the additional burden on caregivers (39). Fully electronic formats may be more user-friendly, less time-consuming, and associated with fewer errors (40). Moreover, the use of structured, researcher-led interviews could improve data quality and reduce the risk of missing data in future trials (39). Although the Rome Foundation Pediatric Subcommittee on Clinical Trials (20) recommends the use of daily electronic diaries with reminder alarms to minimize recall bias, weekly recall of pain intensity were used in this study. This approach was chosen to reduce the burden on parents and children and to maintain consistency with other symptom assessments.

While quadruple blinding was originally planned, one research dietitian who enrolled participants and developed individualized meal plans remained unblinded. This was necessitated by the study design, limited research staff, and ethical consideration. However, this may have posed a risk of conscious or unconscious influence, potentially affecting group assignment, participant behavior, and data collection. Future studies may mitigate this risk by dividing responsibilities between an unblinded research team (responsible for diet delivery) and a blinded team (responsible for recruitment, data collection, and analysis).

Importantly, these challenges were likely exacerbated by the lingering effects of the COVID-19 pandemic. Reduced caregiver engagement, disrupted school schedules, and general fatigue with structured monitoring requirements following prolonged restrictions may have contributed to poor adherence and missing data. The pandemic also led to lasting changes in school infrastructure and catering policies, which limited the feasibility of storing or reheating meals and further complicated dietary implementation during school hours.

## 5 Conclusions

This early-terminated RCT did not demonstrate a significant benefit of the low-FODMAP diet in increasing the proportion of responders among children with IBS and FAP-NOS. While a greater mean reduction in abdominal pain intensity was observed in the low-FODMAP diet group, suggesting that the diet may have a modest symptom-relieving effect, the study was underpowered. The small sample size and substantial rate of missing data preclude drawing definitive conclusions regarding efficacy.

Given these limitations, routine implementation of the full low-FODMAP diet in clinical practice should be approached with caution, particularly outside specialized settings. Future studies are needed to evaluate not only the long-term effectiveness of this dietary strategy but also its safety, psychological impact, and influence on the child's relationship with food.

## Data Availability

The original contributions presented in the study are included in the article/Supplementary Material, further inquiries can be directed to the corresponding author.
