# Supplementary material for: Efficacy of a low-FODMAP diet in children with irritable bowel syndrome and functional abdominal pain–not otherwise specified: a randomized controlled trial with early termination
Source: Front Pediatr. 2026 Mar 18;14:1809053. doi: 10.3389/fped.2026.1809053 (PMC13038862; doi:10.3389/fped.2026.1809053)
Supplement: Supplementary file 1 [file Table1.docx]

**S1 Table. Results for secondary outcomes (the available case analysis)**

|  | Low-FODMAP diet | | Regular diet | | MD/RR (95% CI) | p |
| --- | --- | --- | --- | --- | --- | --- |
|  | n |  | n |  |  |  |
| **Responders in stool consistency (who met pre-specified criteria for stool consistency improvement),* n (%)** | | | | | | |
| w4 vs. baseline |  |  |  |  |  |  |
| Yes | 13 | 2 (15.4) | 13 | 3 (23.1) | 0.7 (0.1;3.4) | 0.776^1^ |
| No |  | 4 (30.8) |  | 5 (38.5) |  |  |
| Normal |  | 7 (53.8) |  | 5 (38.5) |  |  |
| **Change in abdominal pain frequency from baseline (reported as number of participants with decline, increase or and no change),** n (%)** | | | | | | |
| w1 vs. baseline |  |  |  |  |  |  |
| Decline | 18 | 8 (44.4) | 14 | 6 (42.9) | 1.0 (0.5;2.3) | 0.531^1^ |
| Increase |  | 5 (27.8) |  | 2 (14.3) |  |  |
| No change |  | 5 (27.8) |  | 6 (42.9) |  |  |
| w2 vs. baseline |  |  |  |  |  |  |
| Decline | 17 | 10 (58.8) | 15 | 7 (46.7) | 1.3 (0.6;2.5) | 0.881^1^ |
| Increase |  | 2 (11.8) |  | 2 (13.3) |  |  |
| No change |  | 5 (29.4) |  | 6 (40.0) |  |  |
| w3 vs. baseline |  |  |  |  |  |  |
| Decline | 9 | 7 (77.8) | 12 | 6 (50.0) | 1.6 (0.8;3.0) | 0.314^1^ |
| Increase |  | 0 (0.0) |  | 3 (25.0) |  |  |
| No change |  | 2 (22.2) |  | 3 (25.0) |  |  |
| w4 vs. baseline |  |  |  |  |  |  |
| Decline | 13 | 9 (69.2) | 13 | 4 (30.8) | 2.3 (0.9;5.5) | 0.053^1^ |
| Increase |  | 0 (0.0) |  | 4 (30.8) |  |  |
| No change |  | 4 (30.8) |  | 5 (38.5) |  |  |
| **Change in GSRS total score** | | | | | | |
| w1 vs. baseline, median (Q1;Q3) | 18 | -8.5 (-15.0;0.5) | 14 | -6.0  (-8.0;-3.3) | -2.5 (-9.0;8.0) | 0.834^2^ |
| w2 vs. baseline, median (Q1;Q3) | 17 | -10.0 (-14.0;-3.0) | 15 | -10.00 (-13.5;-5.5) | 0.0 (-6.0;8.0) | 0.910^2^ |
| w3 vs. baseline, mean±SD | 9 | -11.4±12.8 | 12 | -12.3±15.9 | 0.8 (-12.3;13.9) | 0.899 |
| w4 vs. baseline, mean±SD | 13 | -11.0±11.8 | 13 | -8.7±7.9 | -2.3 (-10.5;5.9) | 0.564 |
| **Change in KIDSCREEN-10 index total score [points], mean±SD** | | | | | | |
| w1 vs. baseline | 17 | 3.5±4.3 | 14 | 1.1±5.2 | 2.4 (-1.2;6) | 0.180 |
| w2 vs. baseline | 16 | 1.4±6.6 | 15 | 1.7±5.2 | -0.2 (-4.6;4.1) | 0.915 |
| w3 vs. baseline | 9 | 0.8±6.6 | 12 | 1.1±6.0 | -0.3 (-6.2;5.6) | 0.914 |
| w4 vs. baseline | 13 | 1.2±5.7 | 13 | 0.0±4.6 | 1.2 (-3.1;5.4) | 0.576 |
| **Change in WHO-5 total score [points]** | | | | | | |
| w1 vs. baseline, mean±SD | 17 | 1.5±5 | 14 | 0.1±4.1 | 1.4 (-1.9;4.7) | 0.397 |
| w2 vs. baseline, median (Q1;Q3) | 16 | 2.0 (-0.3;5.3) | 15 | 1.0 (-1.0;3.0) | 1.0 (-2.0;5.0) | 0.341^2^ |
| w3 vs. baseline, median (Q1;Q3) | 9 | 2.0 (1.0;8.0) | 12 | 2.0 (0.8;2.3) | 0.0 (-3.0;6.0) | 0.719^2^ |
| w4 vs. baseline, median (Q1;Q3) | 13 | 4.0 (1.0;6.0) | 13 | 1.0 (-5.0;2.0) | 3.0 (-1.0;7.00) | 0.072^2^ |
| **Change in percentage of child’ days of absence at school [days], median (Q1;Q3)** | | | | | | |
| w1 vs. baseline | 18 | 0.0 (-1.0;0.0) | 14 | 0.0 (0.0;0.0) | 0.0 (-1.0;0.001) | 0.140^2^ |
| w2 vs. baseline | 17 | 0.0 (-1.0;0.0) | 15 | 0.0 (-0.5;0.0) | 0.0 (-1;0.001) | 0.406^2^ |
| w3 vs. baseline | 9 | 0.0 (-1.0;0.0) | 12 | 0.0 (-1.0;0.0) | 0.0 (-1.0;1.0) | >0.999^2^ |
| w4 vs. baseline | 13 | 0.0 (-1.0;0.0) | 13 | 0.0 (0.0;0.0) | 0.0 (-1.0;0.0) | 0.176^2^ |
| **Change in percentage of parents’ days of absence at work [days], median (Q1;Q3)** | | | | | | |
| w1 vs. baseline | 18 | 0.0 (0.0;0.0) | 14 | 0.0 (0.0;0.0) | 0.0 (-0.0;0.0) | 0.787^2^ |
| w2 vs. baseline | 17 | 0.0 (0.0;0.0) | 15 | 0.0 (0.0;0.0) | 0.0 (-0.0;0.0) | 0.974^2^ |
| w3 vs. baseline | 9 | 0.0 (0.0;0.0) | 12 | 0.0 (0.0;0.0) | 0.0 (-0.0;0.0) | 0.716^2^ |
| w4 vs. baseline | 13 | 0.0 (0.0;0.0) | 13 | 0.0 (0.0;0.0) | 0.0 (-0.0;0.0) | 0.518^2^ |
| **Change in BMI-for-age z-score, median (Q1;Q3)** | | | | | | |
| w4 vs. baseline | 18 | 0.0 (0.0;0.0) | 17 | 0.0 (0.0;0.0) | 0.0 (-0.0;0.0) | 0.451^2^ |
| **Compliance [%], mean±SD** | | | | | | |
| w1 | 18 | 77.9±14.8 | 14 | 72.8±15.7 | 5.1 (-6.1;16.3) | 0.361 |
| w2 | 17 | 73.8±18 | 13 | 75.3±14.9 | -1.5 (-13.9;10.8) | 0.802 |
| w3 | 9 | 67.6±19.2 | 10 | 74.0±17.7 | -6.4 (-24.4;11.6) | 0.461 |
| w4 | 13 | 70.6±18.6 | 11 | 75.4±19 | -4.8 (-20.8;11.2) | 0.538 |
| mean | 17 | 73.9±15.9 | 15 | 70.3±16.2 | 3.5 (-8.1;15.2) | 0.539 |
| **Percentage of compliant participants (≥80% consumption of provided diets), n (%)** | | | | | | |
| No | 17 | 10 (58.8) | 15 | 10 (66.7) | 1.2 (0.5;3.1) | 0.927 |
| Yes |  | 7 (41.2) |  | 5 (33.3) |  |  |
| **Tolerance of the low-FODMAP diet [%], mean±SD** | | | | | | |
| w1 | 19 | 70±21.5 | 14 | 71.0±18 | -1.1 (-15.1;13.0) | 0.879 |
| w2 | 17 | 71.5±18.5 | 13 | 67.8±16.5 | 3.7 (-9.4;16.9) | 0.567 |
| w3 | 9 | 64.9±24.3 | 10 | 68.8±17.7 | -3.9 (-25;17.2) | 0.697 |
| w4 | 13 | 68±23.2 | 11 | 66.2±20.3 | 1.8 (-16.7;20.2) | 0.843 |
| mean | 19 | 70.8±20.0 | 14 | 67.9±16.3 | 2.8 (-10.1;15.8) | 0.658 |
| **Adverse events, n (%)** | | | | | | |
| During 4 weeks | 19 | 2 (10.5) | 18 | 2 (11.1) | 1 (0.2;6.0) | >0.999^1^ |
| **Blinding success, n (%)** | | | | | | |
| During 4 weeks | 19 | 12 (63.2) | 17 | 15 (88.2) | 0.7 (0.5;1.1) | 0.128^1^ |

* Responders were defined as participants who showed improvement in their average stool consistency at week 4 compared with baseline [≥1 higher BSFS in case of IBS-C - point higher on the BSFS in the case of IBS-C, or at least one point lower in the case of IBS-D.

** Abdominal pain frequency was reported each week (everyday, 5-6 times per week, 3-4 times per week, less than 3 times per week, none).

BMI, Body Mass Index; FODMAP, fermentable oligosaccharides, disaccharides, monosaccharides, and polyols; GSRS, Gastrointestinal Symptom Rating Scale; WHO-5, World Health Organization Five Well-Being Index
